# KNEE ARTHROSCOPIC VISIBILITY ALTERATIONS IN OBESE AND NON-OBESE PATIENTS

**DOI:** 10.1590/0102-6720201600S10019

**Published:** 2016

**Authors:** Cássio ZINI, Edmar STIEVEN-FILHO, Fernando Issamu TABUSHI, Carmen Australia Paredes Marcondes RIBAS, Fernanda Marcondes RIBAS, Ana Cristina OPOLSKI, Bruna Olandoski ERBANO

**Affiliations:** Postgraduate Program in Principles of Surgery, Evangelic Faculty of Paraná/University Evangelic Hospital of Curitiba/Medical Research Institute, Curitiba, PR, Brazil

**Keywords:** Knee, Arthroscopy, Obesity

## Abstract

**Background:**

Obesity is a chronic disease and has become the most prevalent public health problem worldwide. The impact of obesity on knee is strong and the BMI is correlated with the different alterations.

**Aim:**

Compare surgical visualization of arthroscopic field in partial meniscectomy in obese and non-obese.

**Method:**

Sixty patients were selected, 30 obese and 30 non-obese who underwent arthroscopic partial meniscectomy. The arthroscopic surgical procedures were recorded and analyzed. For the analysis of visualization was used the Johnson's classification (2000).

**Results:**

Were analyzed 48 men and 12 women, the average age was 42.9 years with BMI between 21.56 to 40.14 kg/m^2^. The distribution of visibility of the surgical field according to the classification was: grade 1 - 38/60 (63.3%); grade 2 - 13/60 (21.6%); grade 3 - 6/60 (10%); grade 4 - 3/60 (5%).

**Conclusion:**

Knee arthroscopy did not show a significant difference in the visibility of arthroscopic field in obese and non-obese patients. Thus, it should not be indicated as the preferred method of diagnostic evaluation of joint changes in these patients.

## RESUMO

**Racional:**

A obesidade é doença crônica e tem se tornado o problema de saúde pública mais prevalente em todo mundo. O impacto dela no joelho é grande e o IMC está correlacionado com as diferentes alterações existentes.

**Objetivo:**

Comparar a visualização do campo videoartroscópico na meniscectomia parcial de joelho em pacientes obesos e não obesos.

**Método:**

Foram selecionados 60 pacientes, sendo 30 obesos e 30 não obesos que realizaram meniscectomia parcial videoartroscópica. Os procedimentos videoartroscópicos foram gravados e posteriormente analisados. Foi utilizada na análise a classificação de visibilidade do campo videoartroscópico de Johnson (2000).

**Resultados:**

Foram analisados 48 homens e 12 mulheres com idade média de 42,9 anos e IMC de 21,56 a 40,14 kg/m^2^. A distribuição da visibilidade do campo cirúrgico foi: grau 1 - 38/60 (63,3%); grau 2 - 13/60 (21,6%); grau 3 - 6/60 (10%); grau 4 - 3/60 (5%).

**Conclusão:**

A artroscopia de joelho não demonstrou diferença significativa quanto à visualização do campo videoartroscópico em pacientes obesos e não obesos. Desta forma, não deve ser indicada como método preferencial de avaliação diagnóstica das alterações articulares nesses pacientes.

## INTRODUCTION

Obesity is a chronic disease characterized by excess of body fat and has become the most prevalent public health problem worldwide and is a multifactorial process that involves environmental and genetic aspects^12^. 

According to statistics from the Center for Disease Control and Prevention (CDC), 64% of Americans are above the overweight. In Brazil the problem is also relevant and 40.6% of the population over 20 years is in overweight, according to statistics from the Brazilian Institute of Geography and Statistics in partnership with the Ministry of Health. 

In a recent study Souza et al. (2015) assessed the worldwide prevalence of obesity and overweight. Brazilian men and women, above 20 years of age, had overweight and obesity rates of 52.5% and 58.4%, respectively, while the corresponding prevalence of obesity were 11.7% and 20.6%^24^.

It is estimated that the prevalence of overweight and obesity in children has increased up to five times in developed countries and up to four in developing countries. In Brazil, the proportion of children and adolescents in overweight also increased from approximately 4.1% to 13.9%^7^.

The obesity epidemic is increasing, and related medical and social problems consequently have also increased. Obesity can lead to mechanical changes and orthopedic complications such as joint pain, osteoarthritis, trauma and joint injuries^25^.

It is known that the menisci are important in the function of the knee joint especially in power transmission, increase of joint congruity and consequent stability. These structures contribute to the equilibrium; however, menisci is involved in stabilization, transmitting rotational forces flexion and extension motion converting them into rotational movements and sliding. It is believed that the menisci act on the lubrication of the joint contributing to the distribution of the synovial liquid, having proprioceptive function and articular cartilage nutrition. The menisci are stabilizers in many handling planes, mainly in the anterior^23^. Menisci absorb about 40-60% by weight in orthostatic position, thus protecting the articular cartilage from the effects of gravity ^1^. Therefore, it is also considered that their absence may promote accelerated joint degeneration^15^.

The menisci are anatomic structures exposed to trauma and aging as all joint. Traumatic injuries of the menisci are commonly caused by twisting and compression of the joint. The most common place for the occurrence of injury is the posterior horn and longitudinal tears are the most common type of injury ^3^. 

Significant associations were demonstrated between increased BMI and meniscal lesions in both genders, including obese and overweight adults ^8^. 

The nature non-invasive and non-destructive of arthroscopy aids in the diagnosis of many types of knee injuries. The objective of this study was to compare the visibility of arthroscopic field in obese and non-obese patients.

## METHODS

This study was done in Sports Traumatology and Arthroscopy Center of Vita Curitiba Hospital and Medical Research Institute of the Evangelical Faculty of Paraná, and approved by the Research Ethics Committee of the Evangelical Beneficent Society of Curitiba, PR, Brazil. Guidelines and regulatory standards for research were followed. All patients submitted to arthroscopy were instructed on the procedure and who agreed to perform was requested the authorization for publishing the results. The length of the procedures was between January 2008 and December 2012.

### Selection and criteria for classification of patients with and without obesity

After suspected meniscal injury by physical examination, it was requested magnetic resonance imaging of the knee. It was considered meniscal injury the presence of linear signal hyperintensity area extending to the articular surface. Was calculated the BMI of patients, dividing them into two groups: non-obese when BMI <30 and obese when BMI ≥30.

**FIGURE 1:**
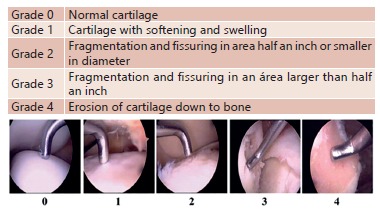
Outerbridge classification^9^

Inclusion criteria were patients who had meniscal injury proven by physical examination and magnetic resonance imaging. Were excluded individuals below 18 years, with concomitant presence of ligament injuries that required surgical repair or reconstruction and chondral lesions grade III and IV according to Outerbridge classification (Figure 1).

### Definition of groups and arthroscopic procedure

Were selected 60 patients, 30 obese and 30 non-obese patients who were submitted to arthroscopic partial meniscectomy in the supine position under general anestesia. There were two horizontal incisions of 0.5 cm for the development of arthroscopic anterolateral and anteromedial portals. Arthroscopic optics with 4 mm in diameter, 140 mm in length and angle of its objective of 30^o^ was introduced through the surgical trocar by traditional anterolateral via arthroscopic feeler via anteromedial for intra-articular thorough assessment and identification of lesions. The arthroscopy of the knee was performed by conventional technique of triangulation. Routine inspection was conducted of the whole joint in all cases, analyzing the patellofemoral joint and its recesses, intercondyle (cruciate ligaments) and finally the medial and lateral compartments.

**FIGURE 2:**
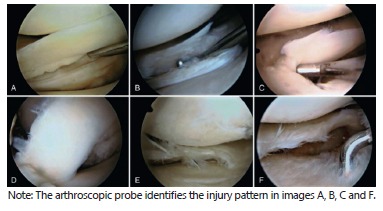
Meniscal tears classification: A) longitudinal tear; B) horizontal tear; C) radial tear of posterior horn; D) bucket handle tear; E) complex tear; F) degenerative lesion without tear.

For evaluation of the medial compartment and medial meniscus, the knee was positioned in flexion 0-60º with valgus stress by minimum abduction of the ipsilateral hip and lateral support of a fixed vertical bar on the edge of the surgical table. The lateral meniscus was visualized with the knee in flexion 90-120º and varus stress produced with external rotation of the ipsilateral hip. After identification and classification of the lesion (Figure 2), the partial resection of meniscus injury was performed piecemeal arthroscopic clamp 3.5x130 mm. This procedure was recorded in digital media and archived along the patient's chart.

### Visibility score rating

**FIGURA 3:**
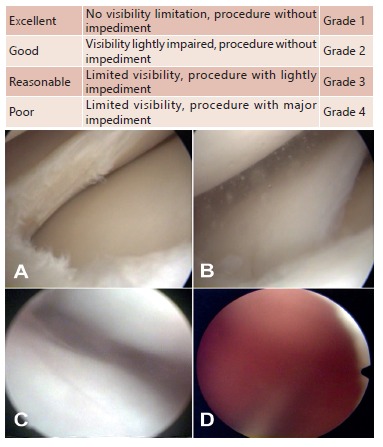
Arthroscopic visibility classification^10^: A) grade 1; B) grade 2; C) grade 3; D) grade 4

The images were evaluated in order to check the visibility through the arthroscopic examination course^27^; considering bleeding, vortexed and image distortion used the visibility score according to Johnson et al.^10^ (Figure 3).

### Statistical analysis

Data were collected in Excel spreadsheets. Normality of distribution was studied by Kolmogorov-Smirnov test. Central tendency measures were expressed as mean and standard deviation if the data were parametric (or Gaussian distribution) and interquartile ranges and median (IQR) when not gaussian. For comparison among groups used the Kruskal-Wallis test for nonparametric data (degree of visibility); significance level was 5% or p=0.05.

## RESULTS

Of the 60 patients submitted to arthroscopy, 34 (56.7%) were on the right side and 26 (43.3%) at the left side. The average age was 42.9 years (18-70). BMI ranged from 21.56 to 40.14 kg/m^2^; median 29.57; IIQ of 25.69 to 35.49 kg/m^2^. In 37 were identified isolated lesions of the medial meniscus (61.6%) and 16 (26.7%) in the lateral meniscus. Were found seven cases of knee injuries in both menisci (11.7%,Table 1).

**TABLE 1:** Meniscal identification

Group	Medial meniscal tear	Lateral meniscal tear	Medial and lateral menisci tears	n
A - Obese	15	8	7	30
B - Non-obese	22	8	0	30

### Analysis of magnetic resonance imaging (MRI)

Through MRI, the meniscal tear pattern was classificated. Table 2 shows the types of lesions found in both groups of patients. MRI diagnosed all meniscal tears, confirmed at the time of arthroscopy.

**TABLE 2:** Meniscal tear pattern

Group / Tear pattern	Longitudinal	Horizontal	Radial	Bucket handle	Complex	Degenerative
A - Obese	9	4	9	4	2	0
B - Non-obese	12	1	9	8	2	0

### Visibility of the videoarthroscopic field 

This visibility is 1 to 4, 1 being the median (IQR 1.0 to 2.0). The distribution of the surgical field visibility was: grade 1 - 38/60 (63.3%); grade 2 - 13/60 (21.7%); grade 3 - 6/60 (10%); grade 4 - 3/60 (5%). Table 3 shows the distribution of degrees of visibility in each group. Was not found grade 4 in the group of non-obese patients.

**TABLE 3:** Visibility classification of arthroscopic field in each group

Group	A Obese	B Non-obese
n	30	30
Visibility using nominal data	Grade 1 - 17/30- 56.6% Grade 2 - 7/30 - 23.3% Grade 3 - 3/30- 10% Grade 4 - 3/30- 10%	Grade 1 - 21/30- 70% Grade 2 - 6/30- 20% Grade 3 - 3/30-10% Grade 4 - 0/30

### Comparison between obese and non-obese

The obese and non-obese patients were compared regarding the degree of visibility and the meniscal tear patterns on MRI. Table 4 shows the comparison for the degree of visibility in continuous variable (p=0.2175) and discrete (p=0.3210). There was no statistical difference between obese and non-obese patients. This result was also observed on MRI images.

**TABLE 4:** Visibility grades between obese and non-obese patients - continuous and discrete variable

	Obese	Non-obese	p
Visibility grade	1 to 4, median 1	1 to 3, median 1	0,2175 Mann-Whitney
Continuous variable	IQR 1 to 2	IQR 1.0 to 2.0	

## DISCUSSION

In this study the mean age was 42.9 years; 80% were men reflecting higher incidence in this gender.

This study included patients of both genders aged between 18-70 years. Sherman et al.^22^ corroborate with this results. Age below 18 years was considered an exclusion criterion. In the study of Camanho^4^ the prevailing average was 50-59 years (34.7%) among 435 patients included, where 261 were male (60%) and 174 female (40%).

Obesity is a risk factor for locomotion activity and induces to cardiovascular, metabolic, endocrine, oncologic, respiratory, liver and bone complications. Following the global trend, the obesity prevalence in Brazil is increasing, which was also observed in our Sports Traumatology and Arthroscopy Center of Vita Curitiba Hospital. Similar data also ws observed by Mendonça^14^.

In 2015 the World Health Organization estimated that the prevalence of approximately 2.3 billion overweight adults and more than 700 million obese worldwide. Considered the chronic disease most prevalent in developed countries, it affects men and women of all races and all ages, reduces the quality of life and has high rates of morbidity and mortality.

Excess weight affects almost the entire body and can lead to numerous complications, among them the degenerative joint disease. Obesity and aging of cartilage cause agility and tissue elasticity loss, and results in decreased joint function, higher frequency of pain and lesions secondary to premature aging of cartilage^13^.

According to Erdil et al.^6^, the obesity epidemic continues to grow worldwide and it is observed that is also associated with orthopedic disorders. Short-term outcomes after arthroscopic partial meniscectomy reflect significant improvement in subjective outcome. However, patients with moderate or significant obesity (BMI>26) have inferior short-term outcomes compared with non-obese. Orthopedic surgeons need to know the impact of obesity on surgical interventions, reason why this research has been developed comparing groups of obese and non-obese patients.

Magnetic resonance imaging demonstrated a high sensitivity and specificity for detecting meniscal injuries. This fact has already been raised by Dorsay and Helms^5^ that showed similar results to identify bucket handle injuries. Although this study did not classified obese patients in grades, Erdil et al.^6^ found that obesity directly causes worsening of joint damage as increases with the degree of obesity.

The resonance proved effective in diagnosis of injuries and is non-invasive. In this study the data obtained from it were not overcome by arthroscopy in their degrees of visibility, as this exam identified all the lesions observed during the arthroscopy and showed no false-negative results. In Magee and Williams^11^ paper, the sensitivity of resonance in the detection of meniscal tears was 96%, and the specificity was 97% compared with arthroscopy in the detection of meniscal tears. It follows that arthroscopy should not be approached as a preferred method for the diagnosis of joint damage in obese.

Arangio and Kostelnik^2^ investigated intra-articular pressure required for adequate knee arthroscopy of the knee. The results showed that arthroscopy could be performed with the knee in any position with a minimum pressure of 55 mmHg. This pressure was adopted in arthroscopic surgical procedures in this research.

According Camanho^4^ the medial and lateral meniscus is injured in 81.8% and 18.2% of cases, respectively. In this research it was found 37 tears of the medial meniscus (61.6%), 16 with lateral meniscus injuries (26.7%) and in seven (11.7%) had both menisci injured. 

In relation to support loads, the menisci absorb about 40-60% by weight in orthostatic position, protecting thus the articular cartilage of the effects of gravity^1^, for which reason the obese patient suffers accelerated joint degeneration and has an additional risk for meniscal lesions^15^.

Rocha et al.^19^ reported prevalence of 67% of meniscal injuries associated with knee ligament rupture, and 35% were lesions of medial meniscus, 14% injuries of the lateral meniscus and 18% of injuries in both menisci. In this paper there was also predominance of injury in the medial meniscus, corroborating with the results of these authors.

The BMI data showed high percentage of the population with overweight, considering BMI of 18-25 kg/m^2^ as normal. In Brazil, studies reported the growing trend of obesity in children and adults over the past decades. International studies have also found high rates of obesity entered in the general scenario of non-communicable diseases. This condition may predispose to several comorbidities, mainly overloading the musculoskeletal structure, such as the knees. The overweight and obesity compared with musculoskeletal discomfort was found in greater numbers in postmenopausal women^28^, different from the results found in this study that showed a higher incidence of males.

The functional results for 1,090 patients who underwent partial meniscectomy, in two different orthopedic clinics, were evaluated retrospectively by Erdil et al.^6^. The study included cases with arthroscopic partial meniscectomy for isolated meniscal tears; patients with concomitant knee disease were excluded. Three hundred forty-one (31%) patients with isolated lateral meniscal tears, 628 (58%) patients with isolated medial meniscal tears, and 121 (11%) patients with both medial and lateral meniscal tears underwent arthroscopic partial meniscectomy. The present research agreed with the incidence of the injured meniscus presenting 61% in medial meniscus, 27% of the lateral and 12% in both menisci.

Salata et al.^20^ believed that loss of meniscal tissue leads to osteoarthritis and poor knee function, many variables may significantly influence this outcome. Four randomized controlled trials, two prospective cohorts, and 23 retrospective cohorts that fit the criteria for levels I, II, and III level of evidence were included in this systematic review. Predictors of poor clinical or radiographic outcomes included total meniscectomy or removal of the peripheral meniscal rim, lateral meniscectomy, degenerative meniscal tears, presence of chondral damage, presence of hand osteoarthritis suggestive of genetic predisposition, and increased body mass index. In this study was found in the 30 obese patients included a total of 15 lesions in medial meniscus, eight in lateral and seven in both.

Several studies have shown that knee lesions are associated with obesity, physical stress at work, traumatic knee injuries, heredity and female gender. Toivanen et al.^26^ prospective study confirms the roles of obesity, heavy work load and knee injury in the etiology of knee lesions. In this study there was a higher frequency of lesions in men.

Despite the multifactorial nature of musculoskeletal disease, obesity consistently emerges as a key and potentially modifiable risk factor in the onset and progression of musculoskeletal conditions of the hip, knee, ankle, foot and shoulder. To date, the majority of research has focused on the impact of obesity on bone and joint disorders, such as the risk of fracture and osteoarthritis. However, emerging evidence indicates that obesity may also have a profound effect on soft-tissue structures, such as tendon, fascia and cartilage. Although the mechanism remains unclear, the functional and structural limitations imposed by the additional loading of the locomotor system in obesity have been almost universally accepted to produce aberrant mechanics during locomotor tasks, thereby unduly raising stress within connective-tissue structures and the potential for musculoskeletal injury. While such mechanical theories abound, there is surprisingly little scientific evidence directly linking musculoskeletal injury to altered biomechanics in the obese. For the most part, even the biomechanical effects of obesity on the locomotor system remain unknown. Given the global increase in obesity and the rapid rise in musculoskeletal disorders, there is a need to determine the physical consequences of continued repetitive loading of major structures of the locomotor system in the obese and to establish how obesity may interact with other factors to potentially increase the risk of musculoskeletal disease^29^, that's why there is the need for studies comparing the articular lesions found in obese and non-obese patients.

In Reigstad e Grimsgaard^18^ study, a total of 876 procedures performed on 785 patients were left for examination. The overall complications rate was low, giving total of 5%. Duration of surgery was the only predicting factor for postoperative complications. Surgical time in this study ranged from 10.40 to 65.20 min.

Salzler et al.^21^ examined the nature and frequency of complications after the most common arthroscopic knee procedures, with particular attention to fellowship training, geographic location of practice, and age and sex of the patient. The authors concluded that knee arthroscopy is not a benign procedure, and patients should be aware of the risk of complications. In this study, the knee arthroscopy was performed with the orientations of the triangulation technique, described by Watanabe^17^.

Ozkoc et al.^16^ did a study to define the clinical features and characteristics of radial tears in the root of the posterior horn of the medial meniscus and to report the outcome of arthroscopic treatment. Arthroscopic meniscus surgery was performed on 7,148 knees. Of those, 722 (10.1%) were radial tear in the root of the posterior horn of the medial meniscus. That type of meniscal tear is strongly associated with obesity and older age and is morphologically different from the degenerative tears that often occur in the posterior horn. In this research, tears observed in obese patients were also more frequent in the medial meniscus but with a complex pattern, according to the morphological classification.

Meniscal tears are common knee injuries, with limited reported data on associated factors, let alone risk factors. Significant associations were demonstrated between increasing BMI and meniscal surgeries in both genders, including obese and overweight adults^9^. The same relationship was found in this research.

## CONCLUSION

Knee arthroscopy did not show a significant difference in the visibility of arthroscopic field in obese and non-obese patients. Thus, it should not be indicated as the preferred method of diagnostic evaluation of joint changes in these patients.
